# Implant Prosthetic Rehabilitation with Bone Regenerative Techniques after Fracture of the Upper Central Incisors

**DOI:** 10.1155/2013/387206

**Published:** 2013-05-12

**Authors:** Massimo Amato, Vincenzo Bruno, Giuseppe Pantaleo, Antonio Cerutti, Gianrico Spagnuolo, Gilberto Sammartino

**Affiliations:** ^1^Department of Medicine and Surgery, University of Salerno, Fisciano, 84084 Salerno, Italy; ^2^Department of Neurosciences, Reproductive and Odontostomatological Sciences, University of Naples Federico II, 80131 Naples, Italy; ^3^School of Dentistry, University of Brescia, 25121 Brescia, Italy

## Abstract

A case of implant-bone prosthetic rehabilitation, after the fracture of the maxillary central incisors, which had been treated with grafting of a bone substitute, is reported. This case was followed by the normal procedures of implantology within the traditional timeframe for bone regeneration. However, a barrier membrane was not used which shows that even along with the use of graft material a sufficient amount of bone could be achieved for a subsequent rehabilitation. Therefore, after a five-year follow-up period, osseointegration was maintained with no marginal bone loss.

## 1. Introduction

Dental trauma could be the most common uncomfortable distress of the facial region. Dental coronal fractures and dislocations occur frequently, and because of their position, the teeth of the anterior maxillary area are therefore more usually subjected to this type of injury [1]. Following dental trauma, psychological stress along with the physical pain has been suggested as the main causes of discomfort for the patient, in particular if there is an extensive loss of coronal structure. The restoration of the anterior maxillary teeth and especially of fractured central incisors represents a key intervention for the dentist which could be challenging [2].

## 2. Case Presentation

A 39-year-old caucasian man was referred to the Dental School of the University Federico II of Naples in 2008. His main complaint was localized pain in region 1.1 and 2.1 during the function, the clinical examination showed that either the two fixed prosthesis elements where of ceramic metal as well as the presence of a secreting fistula in correspondence of the mucosa in the apical area of 1.1. A periapical radiograph was performed and the medical history was documented which has no relevance with regard to the disease in question. Dental history reports that prior treatments and retreatments on these teeth have been completed before the prosthesis.

**Figure 1:**
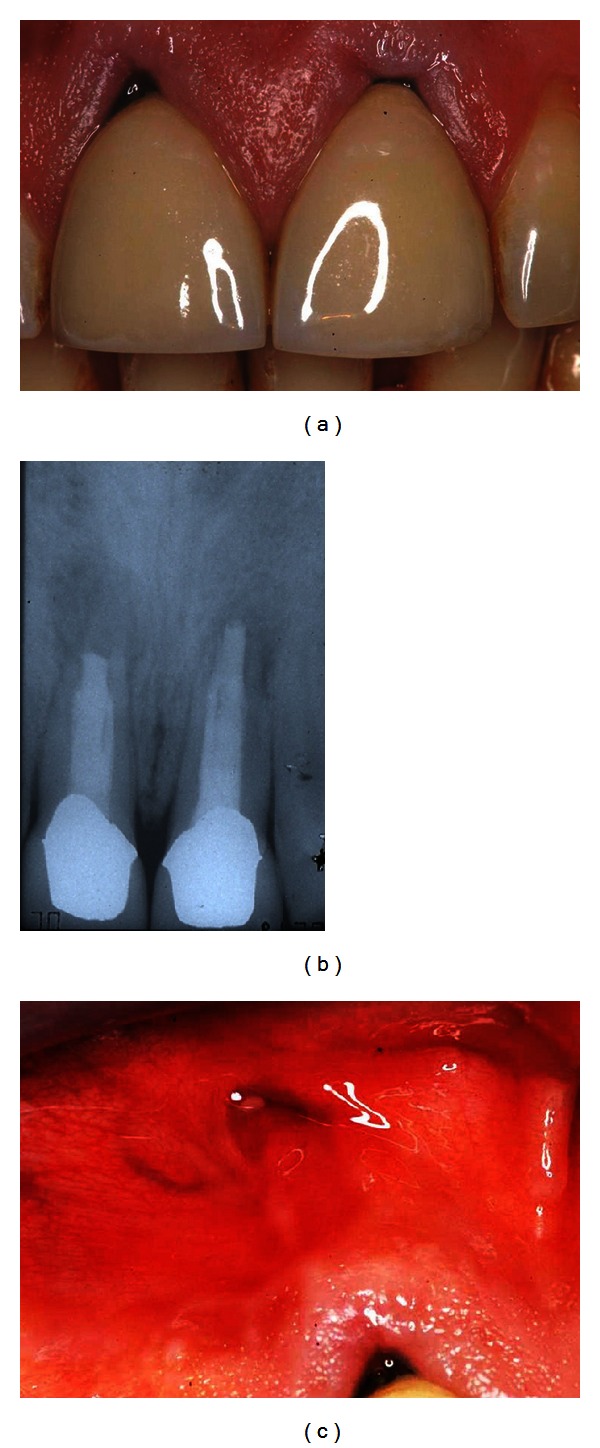
Radiographic (a) and clinical control (b) with the presence of a secreting fistula in correspondence with the mucosa in the apical area of 1.1 (c).

The clinical and radiographic findings showed a probable root vertical fracture of the tooth 1.1 as well as a possible vertical fracture of the tooth 2.1 (Figure 1).

For aesthetic reasons, an alginate impression was taken and a plaster cast was made to construct an interim prosthesis.

Therefore, a decision was made to place two commercial teeth in a row on a steel wire to construct a prosthesis similar to the Maryland Bridge.

Probable causes were an inadequate apical seal and/or one or more vertical root fractures that were not captured via periapical radiograph.

The periapical radiograph from 1.1 revealed an osteolytic area in the middle third of the root. This osteolytic area also enveloped apical third of the root. 

A beta-lactam antibiotic (Amoxicillin) was given orally 2 gr one hour before the surgery, and a diagnostic flap surgery was planned, which allowed to establish the correct treatment plan.

**Figure 2:**
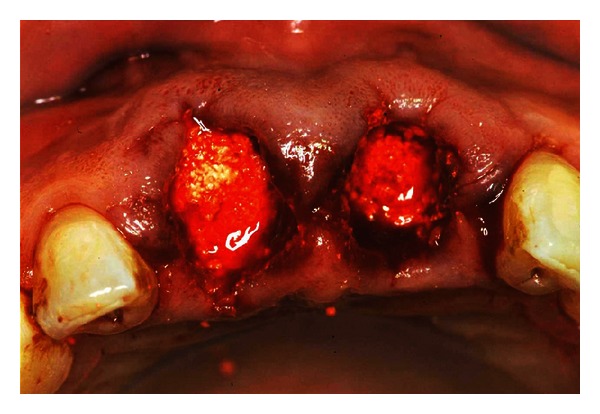
Filling of granules of bone substitute (Bio-Oss spongiosa) into the socket.

Previous to local anesthesia, a diagnostic flap was opened, respecting marginal soft tissues. The root's vertical fracture of 1.1 was immediately evident, while curettage of the exposed portion of the root of 2.1 also revealed a further fracture in the apical mesial portion of the root. The teeth were gently extracted with particular attention to the preservation of the hard and soft tissues at the sites. The extraction sockets were debrided using piezosurgery devices and alveolar surgical curettes to remove the granulation tissue [3]. The socket walls were then carefully probed to assess the presence of any fenestration or dehiscence defects [2]. Atraumatic avulsion was required to maintain the integrity of the vestibular bone, which specifically in the frontal region is as usual particularly thin. The preservation of the buccal bone is extremely important to obtain a good esthetic outcome in the frontal area and medium- and long-term maintenance [4]. If any trauma were to occur to the buccal bone crest, it would hinder the recovery and cause additional damage. The extraoral view of the roots confirmed the diagnosis. The alveolar bone review showed a defect of 8 mm. According to the conclusion of Darby et al., 2009, a decision was made to adopt a technique for ridge preservation [1, 5]. In particular, the circumferential gap was filled with granules of bone substitute (Bio-Oss, spongiosa, 0.5 mm). This material has exceptional biological and mechanical properties as well as biocompatibility. Its high porosity provides all the necessary space for angio- and osteogenesis. The microstructure of the Bio-Oss surface promotes optimal proliferation of osteoblasts and particles integrate with the newly formed bone. The slow rate of conversion of Bio-Oss in autologous bone (remodeling) stabilizes the structure of the newly formed to maintain a good long-term bone volume increased (Figure 2) [6, 7].

**Figure 3:**
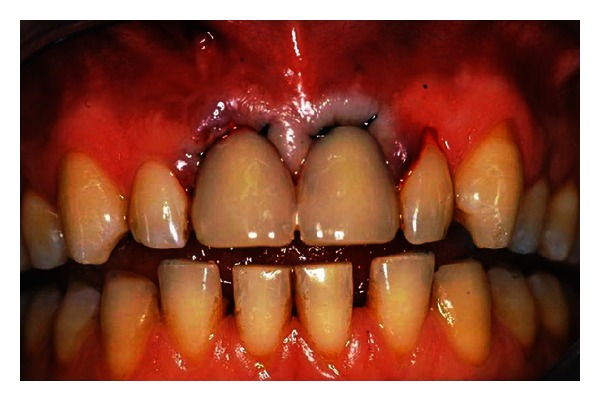
Temporary adhesive system attached to the adjacent teeth.

The Vicryl polyglactin (91, 3/0) absorbable suture was used to close the flap. The interim prosthesis was delivered by using an adhesive system to attach to the adjacent teeth. This prosthesis allows to achieve an acceptable esthetic outcome, as well as a good phonatory function and an initial tissue conditioning for the pontic areas (Figure 3) [8]. 

The postoperative therapy requires good oral hygiene, rinsing with mouthwash containing 0.2% chlorhexidine solution twice a day, and an evening application of the same product in gel form, as well as the administration of a nonsteroidal anti-inflammatory aid (Ketoprofen 80 mg) for three consecutive days.

The patient was asked to be seen for regular followups for the next three months.

**Figure 4:**
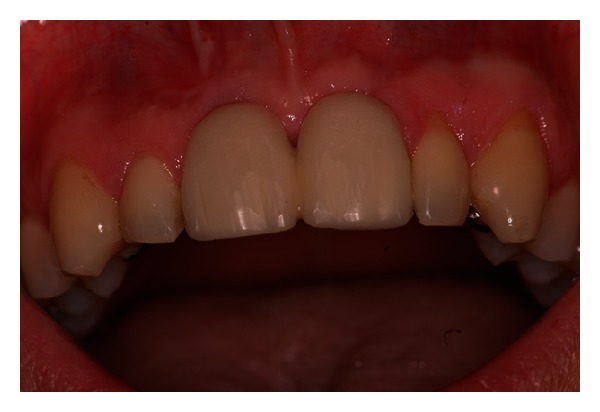
Clinical control at three months. The patient showed remarkable healing of the soft tissues, and the gingiva appeared with an excellent color and texture of the tissue too. It also began to outline the proper and harmonious design of the facial mucosa curvatures, which were conditioned by the interim prosthesis.

At the end of the 3 months, the patient showed remarkable healing of the soft tissues, and the gingiva appeared with an excellent color and texture of the tissue too. It also began to outline the proper and harmonious design of the facial mucosa curvatures, which were conditioned by the interim prosthesis (Figure 4).

**Figure 5:**
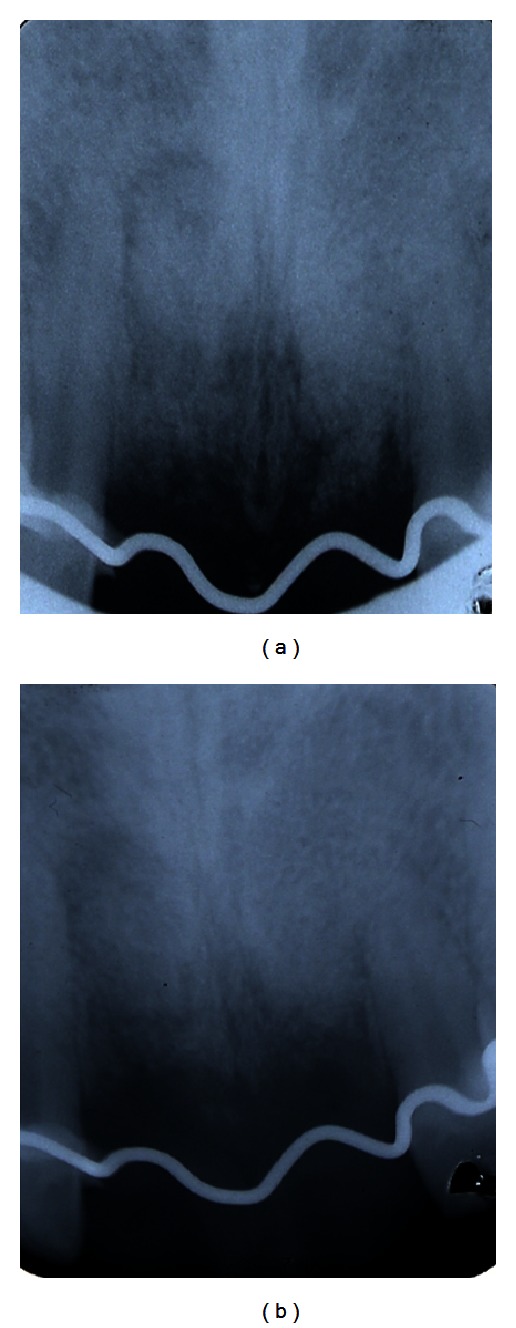
A periapical radiograph was taken at 3 (a) and 6 months (b) from the socket preservation. After 6 months, a limited bone loss was showed related to the steel wire of the interim prosthesis.

At six months, from the socket preservation, a periapical radiograph was taken and showed limited bone loss, which was related to the steel wire of the interim prosthesis (Figure 5).

Moreover, radiographically, the bone appeared homogenous and with a good radiopacity to show a sufficient remineralization.

**Figure 6:**
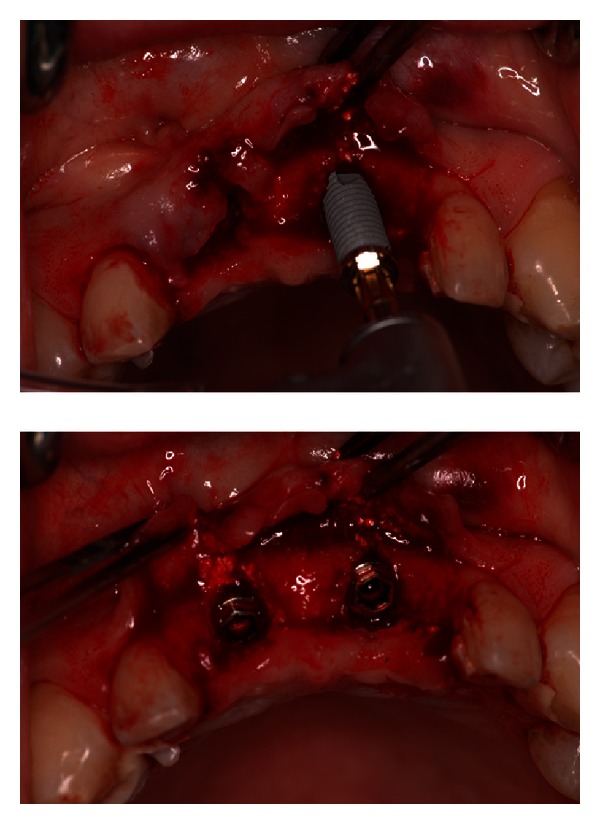
Two 13 mm long implants with a 3,75 mm diameter were placed.

An alginate impression with the interim bridge in situ was taken, to construct a surgical template. The patient was satisfied with regard to the esthetic outcome. A thermoplastic vacuum-formed template was then built and two holes corresponding to the cingulum of the maxillary central incisors were performed. The temporary bridge was removed to place two implants. A bone trephine was used to obtain a bone specimen to assess the bone quality and structure. The implant phase, however, was necessary to find the maximum stability in the apical zone [9]. In the presented case, the extracted roots were 11 mm long, and therefore two 13 mm long implants were placed with a 3,75 mm diameter (Nobel Biocare Branemark System) (Figure 6).

After the initial surgical phase, two resin temporary crowns were applied leaving a sufficient space to allow for any swelling. Only after three weeks, the interim crowns were modeled with a slight contact to the soft tissue.

At six months, the implants were uncovered and a resin screw retained prosthesis was constructed with an emergence profile which was suitable for supporting the buccal soft tissue. It also important to remember to avoid any excessive compression, which could lead to a soft tissue shrinkage in the subsequent months of maturation. The occlusal contacts were verified for the presence of a slight contact during maximum intercuspation using shimstock (Almoreshimstock, 8 mm wide, 8 mm thick) in order to protect the implants from any intense forces. Of course, functional loads resulting from lips, tongue, and food bolus will remain [10, 11].

After waiting for another 6 months, we proceeded to the prosthetic finalization. The patient has a good soft tissue maturation induced by the design of temporary crowns. A periapical radiograph was taken to evaluate the proper bone remodeling.

A custom impression of the healed soft tissue, recording and transferring the soft tissue contour with a gingival outline, was taken according to the technique of Hinds, 1997 [12].

Therefore, after trying all the components, the definitive gold-ceramic crowns were delivered with good accuracy as well as a proper emergence profile to support the tissue. 

The patient was instructed to maintain good oral hygiene by brushing and flossing.

A year later, the patient was seen to observe overall tissue healing, aesthetics, and radiographic osseointegration of the implants.

**Figure 7:**
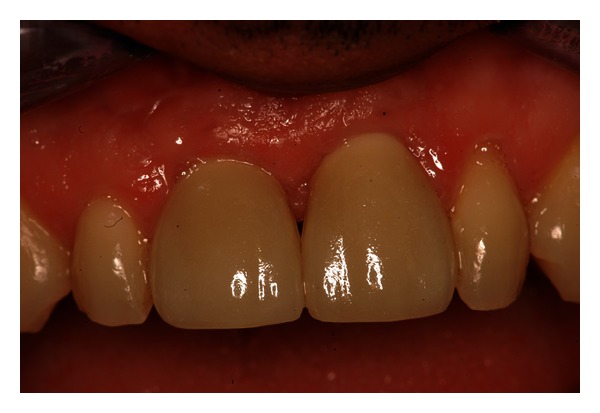
Clinical control after 1 year from the surgery.

This type of rehabilitation used integrates in the morphological and occlusal context of the oral cavity. In this way, this procedure, appropriately managed, is capable of producing the desired implant-prosthetic outcome, ensuring comfort and patient satisfaction (Figure 7) [13].

We recommended the patient a thorough oral hygiene through the use of brush, dental floss, and pipe cleaner.

**Figure 8:**
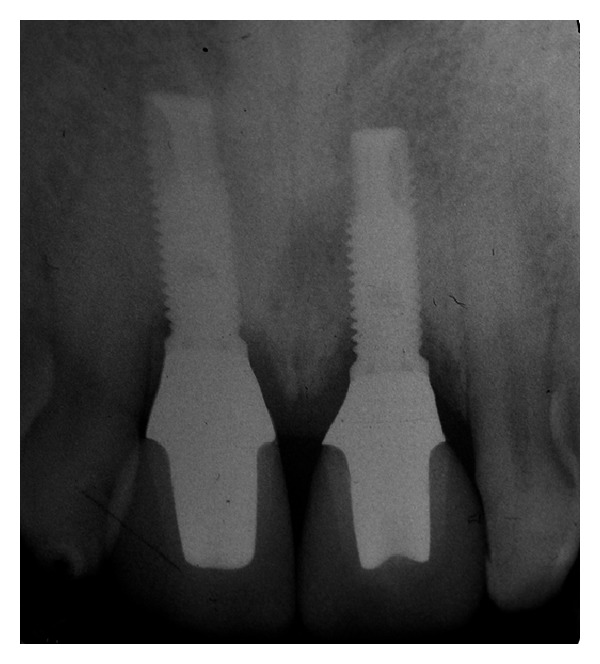
Periapical radiograph after 5 years which showed osseointegration of the implants with only a minimal loss of bone height according to the Albrektsson criteria.

The patient was recalled for a clinical control after a three- and five-year period. A new periapical radiograph was taken which showed an excellent osseointegration of the implants with only a minimal loss of bone height according to the Albrektsson criteria and ICOI Consensus Conference, 2007 (Figure 8) [14, 15].

Overall the patient was quite satisfied with the esthetic outcome and had no clinical issues.

## 3. Discussion

With regard to implant-prosthetic rehabilitation after fracture of the upper central incisors, various techniques have been proposed, which include the postextractive immediate loading implant. In this particular case, however, we felt it was necessary to wait for bone healing after the use of a substitute bone graft, without a barrier membrane. In addition to chemical and physical properties of the material, the aesthetic success must not be underestimated.

Moreover, the primary stability of implants was apically searched in the residual native bone and the major axes were placed palatally, in correspondence with the cingulum to maintain the possibility to build a screw-retained crown and to preserve the height and thickness of the facial bone wall. Therefore, the implant shoulder was positioned about 1 mm palatal to the point of emergence at the adjacent teeth. When the implant is placed too facially, a resorption of the facial bone wall could occur with a subsequent recession. With the implant positioned too palatally, an implant crown with a ridge-lap design might be needed.

The position of the implant must be chosen by using a 3D position, which respects the comfort zone, as stated by Belser et al. [16]. Therefore, the key issues were then the distance of the implants from the adjacent teeth, as well as the orofacial and the apicocoronal position. Care must be taken in the mesiodistal remaining space between the implant and the adjacent teeth: a minimum of a 1.5 mm space must be left for the maturation of the papilla. The formation of the papilla does not depend on the implants, but on the underlying bone support; in fact, soft tissue follows the bone in its process of remodeling. It has been shown that the average thickness of soft tissue is 4.3 mm ± 1 mm. The Authors observed that the presence of the papilla depends on the distance between the bone crest and the contact point: when this distance is ≤5 mm there will always be a complete maturation of the papilla. It has been shown that, when this distance increases to 6 mm, in 56% of cases this will cause an opened papilla with the presence of the classic “black hole” (Tarnow et al. 1992) [17]. Therefore, when making a prosthetic restoration on implants in the frontal area, the contact point must be brought as apically as possible, to avoid the loss of soft tissue. At the same time, when the mesiodistal distance between the tooth and the implant is less than 1.5 mm, there will be loss of the papilla in that area. Equally, a minimum distance of 3 mm between the two adjacent implant's emergences was required [18].

Therefore, prosthetic phase is extremely important.

In this case, we could ask if a barrier it is necessary for the alveolar ridge preservation and the answer is indeterminate at this time. The results between the studies regarding the use of any barrier are almost similar; no clear benefit was demonstrated with or without a barrier. Use of a barrier may prove beneficial in cases where extraction socket walls are partially or completely missing [19, 20].

**Figure 9:**
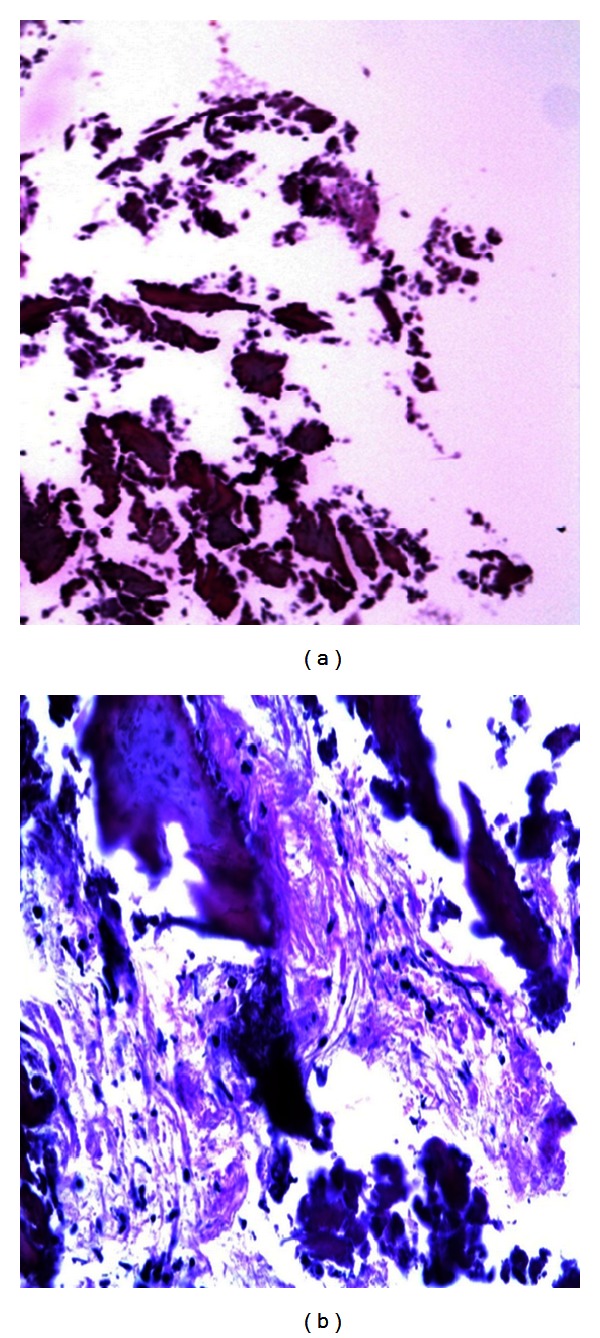
(a) Microscopic findings of implanted bone showing fragments of bone embedded in fibrous tissue (hematoxylin and eosin 106x). (b) The same fragments at higher magnification showing fibroblasts, calcifications, fragments of bone tissue, and traces of grafted material (hematoxylin and eosin 430x).

To make sure of obtaining a good bone growth, we have performed a sample of bone from the site analyzing it histologically. Microscopic findings of implanted bone showed fragments of bone embedded in fibrous tissue; the same fragments at higher magnification showed fibroblasts, calcifications, fragments of bone tissue, and traces of grafted material (Figure 9) [21].

However, additional controlled and comparative studies are needed to confirm or refute these findings.

Another important matter is whether the addition of growth factors could provide a benefit for alveolar ridge preservation procedures [19].

However, a small number of studies have evaluated the use of growth factors for alveolar ridge preservation after tooth extractions, and there was significant heterogeneity in the condition of the host extraction socket and bone at time of graft placing [22]. Use of growth factors may give benefits in cases of extreme alveolar defects after tooth extractions, but more studies have to confirm that. Lastly, the cost/benefit ratio of these products must be considered.

## 4. Conclusion

In this report, it has been showed how graft material can be used in extraction sockets without a barrier membrane ensuring nevertheless a good support for the implant prosthetic rehabilitation. Inspite of the nonuse of membranes, the target was obtained, so the question we asked for is that using a grafting material not completely resorbable or with a slow resorption as Bio-Oss, in this technique of resorption prevention, might suggest that the use of membrane does not affects the result with the biological and financial savings of a membrane.

In agreement with literature, our case also shows, that with regards to socket preservation, bone loss was limited without affecting the clinical outcome. Therefore, this specific technique could also be used in an aesthetic area that may be a challenge. Our results show that over a period of time the clinical outcome remained stable.
